# An iron-based beverage, HydroFerrate fluid (MRN-100), alleviates oxidative stress in murine lymphocytes *in vitro*

**DOI:** 10.1186/1475-2891-8-18

**Published:** 2009-05-02

**Authors:** Mamdooh Ghoneum, Motohiro Matsuura, Sastry Gollapudi

**Affiliations:** 1 Charles Drew University of Medicine and Science, Department of Otolaryngology, Los Angeles, California 90059, USA; 2 Jichi Medical University, School of Medicine, Department of Infection and Immunity, Tochigi, 329-0498, Japan; 3 University of California, Irvine, Division of Basic and Clinical Immunology, Irvine, California 92697, USA

## Abstract

**Background:**

Several studies have examined the correlation between iron oxidation and H_2_O_2 _degradation. The present study was carried out to examine the protective effects of MRN-100 against stress-induced apoptosis in murine splenic cells *in vitro*. MRN-100, or HydroFerrate fluid, is an iron-based beverage composed of bivalent and trivalent ferrates.

**Methods:**

Splenic lymphocytes from mice were cultured in the presence or absence of MRN-100 for 2 hrs and were subsequently exposed to hydrogen peroxide (H_2_O_2_) at a concentration of 25 μM for 14 hrs. Percent cell death was examined by flow cytometry and trypan blue exclusion. The effect of MRN-100 on Bcl-2 and Bax protein levels was determined by Western blot.

**Results:**

Results show, as expected, that culture of splenic cells with H_2_O_2 _alone results in a significant increase in cell death (apoptosis) as compared to control (CM) cells. In contrast, pre-treatment of cells with MRN-100 followed by H_2_O_2 _treatment results in significantly reduced levels of apoptosis.

In addition, MRN-100 partially prevents H_2_O_2_-induced down-regulation of the anti-apoptotic molecule Bcl-2 and upregulation of the pro-apoptotic molecule Bax.

**Conclusion:**

Our findings suggest that MRN-100 may offer a protective effect against oxidative stress-induced apoptosis in lymphocytes.

## Introduction

Oxidative stress represents the imbalance between the cellular production of oxidants and the capacity of cellular antioxidant defenses to scavenge these oxidants. It is produced in cells by oxygen-derived species which include free radicals and peroxides; it is also produced at a low level by normal aerobic metabolism and nutritional deficiency in trace metal [1,2]. Increasing evidence indicates that oxidative stress is a major inducer of cell death [3]. In this process some of the reactive oxygen species (such as superoxide) are converted into hydrogen peroxide which can cause controlled apoptotic cell death [4,5].

Oxidative stress is associated with many diseases, including chronic inflammation, arteriosclerosis, diabetes, stroke, Alzheimer's and Parkinson's diseases, and aging [6-8]. In addition, nutritional deficiencies like lack of iron have been shown to induce oxidative stress [9,10] and currently affect over 2 billion people worldwide. Thus far, the ability of iron to protect against oxidative stress has only been studied to a limited extent [11].

Iron has the capacity to accept and donate electrons readily, and this characteristic makes it a useful component of cytochromes and oxygen-binding molecules. Recent studies have suggested ferritin is a protectant against oxidative damage in endothelial cells as well as murine and human leukemia cells [11,12].

In the current study, we examined the possible effect of MRN-100 on oxidative stress-induced apoptosis in murine splenic cells. MRN-100 is an iron-based compound derived from bivalent and trivalent ferrates, and it is sold as a beverage in Japan. The results show that MRN-100 attenuates H_2_O_2_-induced apoptosis in splenic cells.

## Materials and methods

### MRN-100

MRN-100 is an iron-based compound derived from bivalent and trivalent ferrates and was prepared in distilled water (DW) with the concentration of Fe^2+ ^and Fe^3+ ^ions at ~2 × 10^-12 ^mol/l. MRN-100 is obtained from phytosin as previously described [13]. MRN-100 was provided by ACM Co., Ltd., Japan.

### Complete medium (CM)

Complete medium (CM) consisted of RPMI-1640, supplemented with 10% fetal calf serum (FCS), 2 mM glutamine and 100 μg/ml of streptomycin and penicillin.

### Animals

C57BL/6 (4–5 weeks old, 20–25 g female mice) were purchased from Harlan Laboratories (Chicago, IL, USA) and from Saitama Experimental Animal Co. Ltd., (Saitama, Japan). The mice were accommodated for 1 week prior to experimentation. Mice were maintained in the animal facility at Charles Drew University of Medicine and Science, Los Angeles, CA, USA and in the Laboratory Animal Center at Jichi Medical University in Japan. Mice were housed 2 per micro-isolator cage and were fed sterilized standard cube pellets and water *ad libitum*. Animal protocols were in compliance with the Guide for the Care and Use of Laboratory Animals in the USA.

### Preparation of splenic lymphocytes

Mice were killed by cervical dislocation. Spleens were removed, teased in CM, and contaminating erythrocytes were lysed with distilled water for 20 seconds. Splenic lymphocytes were centrifuged, and washed three times with HBSS. Cell viability was 95%, as determined by the trypan blue exclusion test. Cells were counted using a hemocytometer and a light microscope, and were re-suspended to a concentration of 10^7 ^cells/ml in CM.

### Experimental protocol

Splenic lymphocytes (1 × 10^6 ^cells/ml) were cultured in CM and divided into four groups: group 1 – cells were incubated with 25 μm hydrogen peroxide (H_2_O_2_) for 16 hrs; group 2 – cells were incubated with MRN-100 (100 μl/ml) for 16 hrs; group 3 – cells were incubated with MRN-100 for 2 hrs and were subsequently exposed to H_2_O_2 _for 14 additional hrs; group 4 – control group, in which cells were incubated with CM alone for 16 hrs.

### Apoptosis by flow cytometry

Splenic lymphocytes were cultured as described above. The percentage of hypodiploid, apoptotic cells was examined by the propidium iodide (PI) technique using flow cytometry. Briefly, splenic cells (1 × 10^6^/ml) were fixed in 70% methanol, re-suspended in the DNA extraction buffer, washed in PBS and incubated with 50 μg/ml of PI for 30 min at room temperature in the dark, and analyzed by FACScan (Becton Dickinson, San Jose, California, USA).

### Cell death detected by trypan blue exclusion

At 16-hrs post-lymphocyte culture, cells from the different groups (H_2_O_2_, MRN-100, MRN-100 + H_2_O_2_, and control [CM]) were treated with trypan blue stain (100 μl/ml) for 5 minutes and counted using a light microscope (Nikon, Tokyo, Japan) and hemocytometer. The percent of dead cells was calculated blindly out of a total of 300 cells by the same investigator.

### Determination of nitric oxide (NO) production

Splenic lymphocytes (1 × 10^6 ^cells/ml) were cultured in the presence or absence of H_2_O_2 _at concentrations of 0–50 μM for 16 hrs. The mouse macrophage cell line, RAW-264.7, was also tested for NO production in the presence or absence of lipopolysaccharide (LPS) as well as H_2_O_2 _at the above-mentioned concentrations. Production of NO was determined as the amount of nitrite, a stable end-product of NO, in the culture supernatant obtained at 48 hr post stimulation. Nitrite was measured by a colorimetric assay using the Griess reagent (1% sulfanilamide and 0.1% *N*-1-naphtylethylendiamine dihydrochloride in 2.5% H_3_PO_4 _solution) [14]. The absorbance at 540 nm was measured and the nitrite concentration was quantified (in μM) using sodium nitrite as the standard in each assay.

### Intracellular calcium (Ca^2+^) flux

The effect of H_2_O_2 _on Ca^2+ ^levels was examined in two models: splenic lymphocytes and the mouse macrophage cell line, RAW264.7 cells. Cellular Ca^2+ ^flux was determined using Screen Quest™ Fluo-8 NW Calcium Assay Kit (ABD Bioquest Inc, Sunnyvale, California, USA) as follows. Splenic lymphocytes (1 × 10^7^/2 ml CM) were cultured for 2 hrs with either PBS (control) or MRN-100 (10% v/v CM) in a 60 mm culture plate (Corning Inc, Corning, New York, USA). The cells were harvested by centrifugation and resuspended with 1.5 ml of Fluo-8 NW dye-loading solution in the kit. The cell suspension was plated 100 μl per well in a 96-well black well/clear bottom plate (Costar, Cambridge, Massachussetts, USA). In the case of RAW264.7 cells, adherent cells (1 × 10^5 ^cells/well) in a 96-well black well/clear bottom plate were cultured with either PBS or MRN-100. At 2 hrs, the supernatant was discarded and 100 μl Fluo-8 NW dye-loading solution was added. The plates were incubated at 37°C for 30 minutes, and then placed at room temperature for another 30 minutes. H_2_O_2 _solution (25 μl/well) was added to a final concentration of 10, 50 or 250 μM. Fluorescence at Ex = 490/Em = 525 nm was measured by SPECTRAmax M5 (Molecular Devices Corp, Sunnyvale, California, USA).

### Western blot analysis

The protein levels of Bcl-2 and Bax were determined using Western blot analysis. Splenic cells were treated as described above. Cells were harvested, washed with cold PBS [10 mM (pH 7.4)], lysed with ice-cold M-PER mammalian protein extraction reagent (Pierce Biotechnology, Rockford, Illinois, USA) for 30 minutes, and centrifuged at 14,000 *g *for 20 minutes at 4°C. The supernatant was collected and protein concentration was determined using BCA protein assay (Pierce Biotechnology). Seventy-five μg of cell lysates were subjected to Western blot analysis by 4%–12% SDS polyacrylamide gel electrophoresis. Membranes were probed using 1:500 anti-Bcl-2 or anti-Bax (BD Bioscience, San Diego, California USA) primary antibody. The washed PVDF membranes were then incubated with 1:2000 dilution of monoclonal secondary antibody. Immunoreactive bands were visualized by using ECL detection system (Amersham, Buckinghamshire, UK). To verify equal protein loading and transfer, the blots were stripped and re-probed with β-actin using an anti-actin rabbit polyclonal antibody. Data are represented as mean ± SEM. The ratio of Bcl-2/Bax was examined using densitometry of each band.

### Statistical analysis

Using the Student's *t-*test, we tested the significance of the difference in the percent of apoptotic splenic cells between treated and control cells. We considered the cutoff of *p *< 0.05 as significant.

## Results

### Apoptosis by flow cytometry

**Figure 1:**
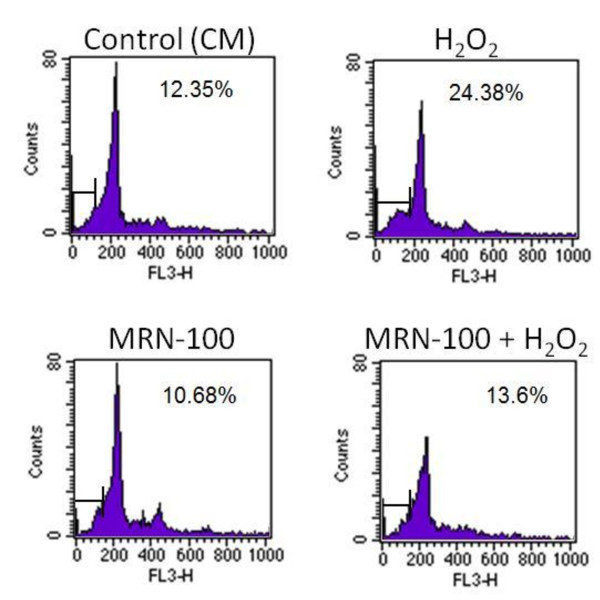
**Flow cytometery analysis of MRN-100-treated splenic lymphocyte apoptosis**. Splenic lymphocytes were cultured in the presence of H_2_O_2_, MRN-100, MRN-100 + H_2_O_2_, or control (CM). The percentage of dead cells was examined by the propidium iodide (PI) technique using flow cytometry. Shown are representative plots from three independent experiments.

Results in Figure 1 show, as expected, that culture of splenic cells with H_2_O_2 _alone results in a significant increase in cell apoptosis as compared to control (CM) cells. In contrast, pre-treatment of cells with MRN-100 followed by H_2_O_2 _treatment results in significantly reduced levels of apoptosis and inhibits the H_2_O_2_-induced apoptosis to the level of control (CM) cells.

### Cell death percentage as examined by trypan blue

**Figure 2:**
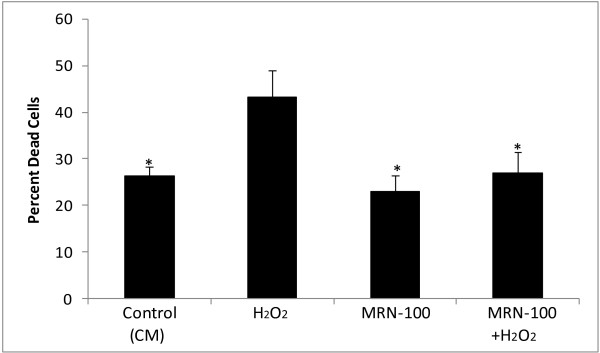
**Cell death analysis of splenic lymphocytes by trypan blue assay**. Splenic lymphocytes were cultured in the presence of H_2_O_2_, MRN-100, MRN-100 + H_2_O_2_, or control (CM). At 16 hrs, cells from the different groups were treated with trypan blue stain for 5 minutes and counted. The percent of dead cells was calculated out of a total of 300 cells. * indicates p < 0.02 as compared to cells treated with H_2_O_2_. Data shown is representative of three independent experiments.

The protective effect of MRN-100 against H_2_O_2_-induced cell death was further confirmed by trypan blue exclusion tests. Results in Figure 2 show a high level of dead splenic cells post-treatment with H_2_O_2_. In contrast, pre-treatment of splenic cells with MRN-100 prevents H_2_O_2_-induced cell death.

### Induction of nitric oxide (NO)

Splenic cells were stimulated with H_2_O_2 _at concentrations of 0–50 μM and nitric oxide (NO) production was examined. Results show that NO production is not detected at any of the concentrations of H_2_O_2 _tested. Similar results were obtained using the mouse macrophage cell line, RAW-264.7; although this cell line produces NO upon stimulation with LPS, NO was not detected following treatment with H_2_O_2 _(data not shown).

### Intracellular calcium

Intracellular Ca^2+ ^was examined in mouse splenic cells and RAW264.7 cells upon stimulation with H_2_O_2_. Results show that an increase of intracellular Ca^2+ ^was scarcely detected in these cells up to 50 μM H_2_O_2 _(data not shown). A high concentration of H_2_O_2 _(250 μM), results in little increase of intracellular Ca^2+^, but MRN-100 pre-treatment shows no suppressive effect (data not shown).

### Bcl-2 and Bax protein level

**Figure 3:**
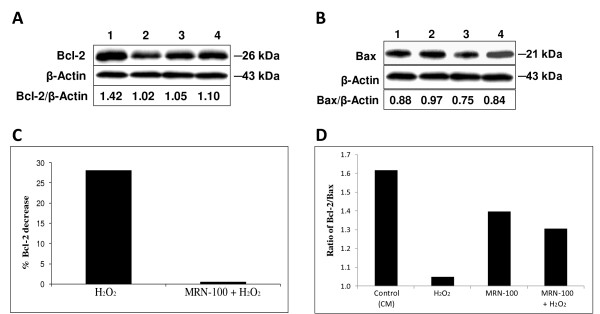
**Effect of MRN-100 on Bcl-2 and Bax protein levels**. Splenic cells were cultured in the presence of H_2_O_2_, MRN-100, MRN-100 + H_2_O_2_, or control (CM). Cell lysates were subjected to Western blot using anti-Bcl-2 or anti-Bax antibodies. Fig. 3A and 3B are representative blots showing Bcl-2 and Bax protein levels, respectively. Lane 1: Control (CM); Lane 2: Splenic cells exposed to H_2_O_2_; Lane 3: Splenic cells treated with MRN-100; Lane 4: Splenic cells pre-treated with MRN-100 and exposed to H_2_O_2_. Depicted below the blots is the ratio of Bcl-2/β-Actin (Fig. 3A) or Bax/β-Actin (Fig. 3B), respectively, as calculated using densitometry. Fig. 3C depicts the percent Bcl-2 decrease (calculated using densitometry) as compared to the respective control: H_2_O_2 _was compared to control (CM), and MRN-100 + H_2_O_2 _was compared to MRN-100 control. The value for H_2_O_2 _treatment was calculated by dividing the value of Lane 2 over the Lane 1 from Fig. 3A. The value for MRN-100 + H_2_O_2 _treatment was calculated by dividing the value of Lane 4 over Lane 3 from Fig. 3A. *highly significant (p < 0.001). Fig. 3D shows the Bcl-2/Bax ratio for each treatment. The Bcl-2 bands were measured using densitometry and were compared to Bax bands (data not shown). The ratio of Bcl-2/Bax is taken from a representative experiment.

The effect of MRN-100 on Bcl-2 and Bax protein levels was determined by Western blot. Bcl-2 is known as an anti-apoptotic protein that has been associated with the inhibition of apoptosis, whereas Bax is a pro-apoptotic protein. Splenic cells were treated for 16 hrs with MRN-100 alone, H_2_O_2 _alone, MRN-100 + H_2_O_2_, or control (CM) cells. The effects of H_2_O_2 _and MRN-100 on Bcl-2 and Bax protein levels in splenic cells are shown in Fig 3A and 3B, respectively. Results show that H_2_O_2 _causes a decrease in protein level of Bcl-2 and an increase in the protein level of Bax in untreated spleen cells (compare lane 1 with lane 2). In contrast, pre-treatment with MRN-100 partially counteracts the effect of H_2_O_2 _on both Bcl-2 and Bax protein levels (compare lane 2 with lane 4). Figure 3C depicts the effect of MRN-100 on H_2_O_2_-induced changes in Bcl-2. The ratio of Bcl-2/Bax was also examined (Figure 3D) by analyzing the densitometry of bands from the Bcl-2 blot (Figure 3A) and Bax blot (Figure 3B). The ratio of Bcl-2 to Bax is reduced significantly in H_2_O_2 _treated spleen cells. Pre-treatment with MRN-100 partially prevents the H_2_O_2_-induced decrease in the ratio of Bcl-2/Bax.

## Discussion

Oxidative stress is caused by the persistent generation of reactive oxygen species (ROS) in cells, which is stimulated by carcinogens, pathogen invasion, inflammation, environmental factors such as toxins and UV stress, nutrients and mitochondrial respiration, and is an inevitable consequence of aging in aerobic organisms [15-17]. Oxidative stress is associated with many human diseases [6-8]. Other studies have shown that oxidative stress dysregulates the functions of immune cells [18,19] and provokes allergies, autoimmune diseases and immune senescence [20,21]. Results of this study show that MRN-100 pre-treatment can protect murine splenic lymphocytes from oxidative stress-induced apoptotic insults. The protective action appears to involve a mitochondria-dependent mechanism.

Recent studies by us and others have shown that oxidative stress induces apoptotic cell death by an intrinsic (mitochondrial) pathway of apoptosis [18,19,22]. This pathway is dependent on the process of mitochondrial outer membrane permeabilization (MOMP) which leads to the release cytochrome C, apoptosis-inducing factor (AIF) and endonuclease G from the mitochondrial intermembrane space into the cytosol [23]. The proteins released from mitochondria induce apoptosis by caspase-dependent and caspase-independent pathways [24]. Mitochondrial membrane permeabilization is regulated by members of Bcl-2 family [24,25]. The pro-apoptotic Bcl-2 family member, Bax, is present in most cells in inactive form, and their activation causes MOMP. The anti-apoptotic molecule Bcl -2 inhibits MOMP by preventing the action of pro-apoptotic molecules [26]. Furthermore Bcl-2 can complex with Bax and it has been suggested that Bcl-2/Bax ratio determines cellular susceptibility to apoptosis [27]. In this study we have shown that MRN-100 inhibits the H_2_O_2_-induced upregulation of Bax and down regulation of Bcl-2. This would suggest that MRN-100 protects splenic cells by maintaining mitochondrial membrane integrity.

The mechanism (s) by which MRN-100 neutralizes the action of reactive oxygen species is not known. MRN-100 is an iron-based compound derived from bivalent and trivalent ferrates. Iron is a known to interact with free radicals. Important chemical reactions through which iron is involved are Fenton reaction and Haber-Weiss reaction [28]. Therefore it is possible that MRN-100 may interact with H_2_O_2 _and prevent its action. Alternatively, MRN-100 may inhibit the H_2_O_2_-induced signaling pathways that culminate in apoptosis. The latter hypothesis is based on the reports which show that H_2_O_2 _induces apoptosis by mobilizing multiple signaling pathways [29,30]. It should be mentioned that in this study we did not observe *in vitro *activation of Ca^2+ ^and NO pathways of apoptosis in splenic cells, suggesting that MRN-100 may alleviate oxidative stress-induced apoptosis in splenic cells by a mechanism other than NO and Ca^2+ ^pathways.

Several studies have examined the correlation between iron oxidation and H_2_O_2 _degradation. Iron has the capacity to accept and donate electrons readily. This capability makes it physiologically essential as a useful component of cytochromes and oxygen-binding molecules. For example, free iron oxidized on clay surfaces efficiently catalyzes the decomposition of H_2_O_2 _[9]. Fe(II) and H_2_O_2 _reaction analysis indicates the participation of further Fe intermediates and, therefore, Fenton redox activities [31]. Iron enhances the protective effect of flavonoids against H_2_O_2_-induced DNA damage [32]. The pre-treatment of several cell lines, including endothelial cells [33], murine and human leukemia cells [34], with acute iron loads which are then exposed to H_2_O_2_, protected the cells against the oxidative damage and various other types of oxidative insults.

The typical American diet may be short in a number of nutrients including iron. Furthermore, the recommended daily allowance (RDA) for many nutrients might be well below what is optimally needed. Iron is incorporated into heme complexes which are typically associated with oxygen carrier proteins. In addition to its ability to protect against oxidative stress, we have shown elsewhere that MRN-100 also possesses anti-HIV activity [13].

## Conclusion

MRN-100, an iron based beverage attenuates oxidative stress-induced apoptosis in immune cells by modulating Bcl-2 and Bax protein levels. Our findings suggest that MRN-100 may have a therapeutic potential in apoptotic disorders associated with excess oxidative stress.

## Competing interests

The authors declare that they have no competing interests.

## Authors' contributions

MG and SG shared designing the experiments and writing the manuscript. MM was responsible for calcium and nitric oxide studies.
